# Oak Species *Quercus robur L*. and *Quercus petraea* *Liebl*. Identification Based on UHPLC-HRMS/MS Molecular Networks

**DOI:** 10.3390/metabo11100684

**Published:** 2021-10-06

**Authors:** Gaëlle Buche, Cyril Colas, Laëtitia Fougère, Emilie Destandau

**Affiliations:** 1 Institut de Chimie Organique et Analytique, Université d’Orléans-CNRS, UMR 7311 BP 6759, CEDEX 2, 45067 Orléans, France; gaelle.buche@univ-orleans.fr (G.B.); cyril.colas@univ-orleans.fr (C.C.); laetitia.fougere@univ-orleans.fr (L.F.); 2 Centre de Biophysique Moléculaire, CNRS-Université d’Orléans, UPR 4301, CEDEX 2, 45071 Orléans, France

**Keywords:** mass spectrometry, molecular network, Venn diagram, sessile oaks, pedunculate oaks

## Abstract

Two species of oak are dominant in French forests: pedunculate oak (*Quercus robur L.*) and sessile oak (*Quercus petraea Liebl*.). Their differentiation is not straightforward but is essential to better understand their respective molecular content in order to better valorize them. Thus, to improve oak species identification, an untargeted UHPLC-HRMS/MS method associated with a two-step data treatment was developed to analyze a wide range of specialized metabolites enabling the comparison of both species of oak extracts. Pooled extracts from sessile and pedunculate oaks, composed of extracts from several trees of pure species from various origins, were compared using first the Venn diagram, as a quick way to get an initial idea of how close the extracts are, and then using a molecular network to visualize, on the one hand, the ions shared between the two species and, on the other hand, the compounds specific to one species. The molecular network showed that the two species shared common clusters mainly representative of tannins derivatives and that each species has specific molecules with similar fragmentation patterns, associated in specific clusters. This methodology was then applied to compare these two pooled extracts to unknown individuals in order to determine the species. The Venn diagram allowed for the quick presumption of the species of the individual and then the species could be assigned more precisely with the molecular network, at the level of specific clusters. This method, developed for the first time, has several interests. First, it makes it possible to discriminate the species and to correctly assign the species of unknown samples. Moreover, it gave an overview of the metabolite composition of each sample to better target oak tree utilization and valorization.

## 1. Introduction

Oak wood is very widespread and used for many applications. Two species are predominant in French forests, sessile oak (*Quercus petraea Liebl*.) and pedunculate oak (*Quercus robur L*.). Some morphological differences, in particular acorns or even leaves, allow foresters to differentiate them in the forest [1]. However, these morphological criteria are not always easy to visualize, especially for coopers, who select the logs they purchase on the roadside. Cooperage turned to a macroscopic anatomical criterion of the wood, the width of tree annual growth or “grain”, which is easily identifiable, and could be correlated with the composition of the wood. Nevertheless, wines and spirits aged in oak barrels with the same grains still present a significant sensory heterogeneity.

Oak barrels are not just containers used for transport but have become an essential tool for aging wines and spirits. The oak used for wine aging modifies the sensory characteristics of wine by releasing various wood compounds, such as aromatic compounds [2,3,4,5], tannins [6,7,8], bitter [9,10], and sweet [11,12,13] tasting compounds into the beverage and by the slow oxygenation process occurring during aging [14,15,16]. The differentiation of species was initially based on tannin content or on the analysis of volatile compounds. Statistically, pedunculate oak is richer in tannin [17], while sessile oak is richer in aromatic compounds [18]. These methods have proven to be unreliable, however, due to the variability of oak composition of individual trees belonging to the same species [17,19,20]. This variability highlights the difficulty of differentiating botanical species based solely on a few chemical parameters.

There are now other reliable methods including genetic analysis [21,22,23], which requires the use of databases and fresh tissue or targeted chemical analyses based on a calculated ratio of quercotriterpenosid (QTT)/glycosylated bartogenic acid (GluBA) content that defines a triterpenoid index used to determine the oak species [24,25]. Other non-targeted analysis methods have been developed to identify metabolite families and highlight discriminating compounds of oak species [26,27]. Still others try to determine the probability of a species’ distribution by non-parametric classification approaches [28]. 

In recent years, molecular networks emerged. This approach consists of organizing and visualizing tandem mass spectrometry data through spectral similarities. Compounds presenting a similar fragmentation pathway are grouped in clusters that highlight the structural relationship between compounds belonging to the same molecular family [29,30]. Crude extracts of various origins containing many metabolites can thus be more quickly characterized by comparing experimental spectral data to each other and to databases. Moreover, molecular networks help to point out compounds of interest in one or several extracts in comparison in order to focus the analysis on these compounds specifically without spending time on known compounds. 

The aim of the present study was to develop a new method able to compare sessile and pedunculate oak samples and to determine the species of unknown samples. Firstly, HHPLCMS/MS analyses of oak wood extracts from sessile and pedunculate oaks were compared using the Venn diagram combined with molecular networking to highlight the differences in profiles between the two species. Secondly, these species profiles were then assessed to determine the species of unknown individual samples due to their distribution between extracts of sessile and pedunculate oaks in the Venn diagram and in the network. 

This methodology allowed for an easier comparison of samples from different species of wood, highlighting the differences in molecular content and a rapid determination of the species for unknown samples supported by the detection of numerous relevant compounds belonging to specific families. Moreover, it affords a better understanding of the molecular composition of the samples that would improve the valorization of oak wood by foresters who cannot determine the species in forests reliably and better target the relevant applications for this content. In addition, it would allow the determination and optimization of the sensory quality of casks, and thus helps cooperage to make a reasoned choice when selecting the type of wood according to the desired sensory characteristics. 

## 2. Results and Discussion

### 2.1. Comparison of Sessile and Pedunculate Oak Chemical Composition

**Figure 1:**
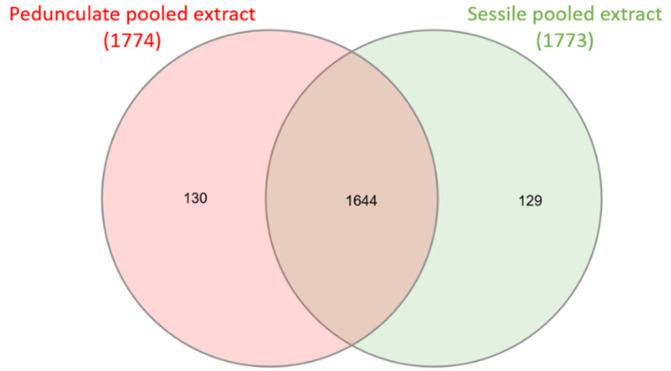
Venn diagram of sessile pooled extracts (21 samples) and pedunculate pooled extracts (21 samples). Numbers above species represent total precursor ions detected in each pooled extract. The circle overlap represents common ions, and the non-overlapping part represents specific ions.

Sessile and pedunculate chemical compositions were first compared using a Venn diagram, presented in Figure 1. 

For both samples, more than 1700 ions were detected in negative ionization mode. The Venn diagram shows that the majority of the ions (1644) are common to sessile and pedunculate oaks, corresponding to molecules related to the genus *Quercus* [31]. Some ions are specific to each of the species, namely, 129 for sessile oaks and 130 for pedunculate oaks. 

In order to correlate this first comparison of the dataset with the mass spectra and to describe the ions that compose it, a molecular network was created using MS/MS analysis of pedunculate and sessile oak samples. The aim was to determine the differences in molecular content between these two species without focusing on a particular molecular family. 

**Figure 2:**
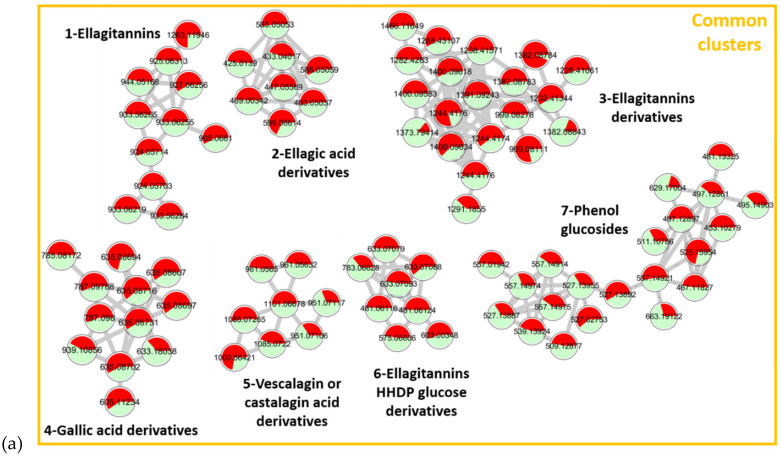

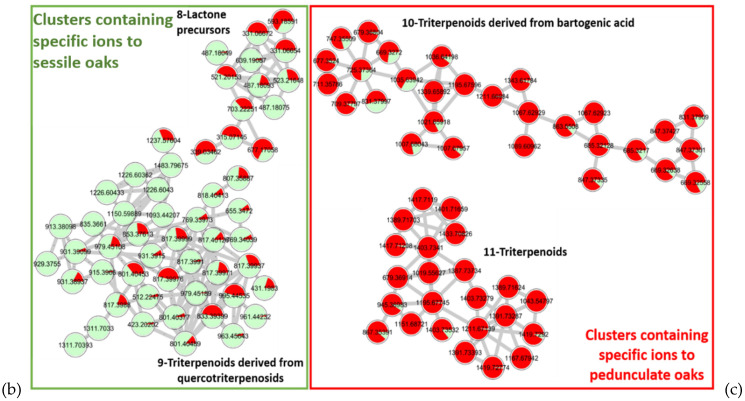
Mapping of pooled extracts (21 samples) of sessile (green) and (21 samples) pedunculate (red) oaks with cos 0.75 and matched fragment 6 parameters. (**a**) Clusters 1–7 representing common clusters between sessile and pedunculated oaks, (**b**) Clusters 8 and 9 containing specific ions to sessile oaks, (**c**) Clusters 10 and 11 containing specific ions to pedunculated oaks.

First, MS/MS spectra were collected and processed on MetaboScape. Then, a quantitative molecular network was generated with Global Natural Product Social Molecular Networking (GNPS) and was visualized using Cytoscape. Molecules generating similar MS/MS spectra are clustered due to similarities in their fragmentation patterns. The resulting molecular networks, presented in Figure 2, visualize chemical relationships of the compounds contained in pedunculate (in red) and sessile (in green) extracts and their relative abundance in the two samples by the proportion of red and green in each node. 

Standards were not available for each cluster, so the clusters were annotated thanks to online databases (PubChem, Lotus, SciFinder, GNPS), the interpretation of the spectra, and the literature. For each cluster, two attempts of annotations are reported in Table 1. Molecules with the same fragmentation pattern and therefore belonging to the same molecular family are associated in the same cluster. As a result, the nodes close to the annotated molecule can be more easily associated with the same type of compounds.

The molecular network shows 11 major clusters. Those numbered from 1 to 7 are composed of ions present in equal amounts in the two oak species, corresponding to the shared molecules highlighted in the Venn diagram. The average retention times of these compounds vary between 3 and 7 min, corresponding to the elution of the most polar compounds in the extracts. Molecules present in the same cluster show common fragment ions that support their association. For each cluster, representative MS/MS spectra are available in supplementary data, Figure S1. 

For Cluster 1, the main similar fragments observed were [C_27_H_19_O_18_]^−^ and [C_14_H_5_O_8_]^−^ corresponding to castalin or vescalin and to ellagic acid, respectively. This cluster includes compounds from the ellagitannin family. 

Cluster 2 is composed of ellagic acid derivatives with [C_14_H_5_O_8_]^−^ as the common fragment of ellagic acids. 

Cluster 3 contains multi-charged ions that have a larger *m/z*. Fragments were identified as vescalagin or castalagin [C_41_H_25_O_26_]^−^, castalin or vescalin [C_27_H_19_O_18_]^−^, and ions corresponding to grandinin or roburin E [C_46_H_33_O_30_]^−^. The molecules in this cluster are assimilated to complex tannins of high molecular weight derived from the ellagitannin family. 

Cluster 4 groups molecules derived from gallic acid. Common losses of 170 mass units corresponding to the loss of gallic acid or even losses of 152 mass units corresponding to galloyl units were observed. 

Cluster 5 shows common losses of 44 mass units corresponding to a decarboxylation, highlighting carboxylic acid functions. Well-known fragments of vescalagin or castalagin and castalin or vescalin were also observed for these compounds of Cluster 5. Losses of 16 mass units (oxygen atom) were observed for deoxy-carboxyvescalagin fragments compared to vescavaloninic acid ones. Cluster 5 groups vescalagin or castalagin acid derivatives.

Cluster 6 is made of molecules built with a unitary brick of ellagic acid [C_14_H_5_O_8_]^−^ and hexahydroxy diphenoyl glucose (HHDP-glucose) [C_27_H_14_O_9_]^−^. This cluster is again a combination of hydrolyzable tannins from the ellagitannin family. 

Cluster 7 groups together compounds with two common ions 183.0655 *m/z* and 313.0574 *m/z*. Depending on the molecules, these fragments are not the same moiety. For 3,4,5-trimethoxyphenyl-(6′-O-galloyl)-O-β-glucopyranoside, the first example in Table 1, 183.0655 *m/z* corresponds to a fragment of trimethoxyphenol, while for 3-methoxy-4-hydroxyphenol 1-O-β-d-(6′-O-galloyl)glucopyranosid, the second example, it is due to a methylgallate moiety. 

The common fragment 313.0574 *m/z* corresponds to the glucogallin moiety (sugar linked to a gallic acid) for the first compound, while it corresponds to the sugar moiety linked to the methoxyhydroxyphenol [Glucogallin−H_2_O−CH_2_] for the second. 

These fragments demonstrate a grouping of compounds formed from a glucogallin and a phenol derivative from the family of phenol glucosides.

All these clusters are common to both species. While pedunculate oaks are often considered to be richer in tannin than sessile oaks, the results obtained show that this cannot be applied to all individuals, since with a pooled sample composed of trees from different origins the mean tannin composition of sessile and pedunculate oaks is quite similar. The high variability of individual tannin composition was already described [17,19,20] and our results confirm that this criterion cannot be used alone to differentiate the two oak species. 

Two clusters clearly appear to be specific to each oak species: Clusters 8 and 9 present compounds specific to sessile oaks while Clusters 10 and 11 present compounds specific to pedunculate oaks. 

Cluster 8 contains ions specific to sessile oak, but unlike the other clusters, which are almost exclusively made of compounds specific to a species, this cluster is nevertheless shared to some extent. Among the specific molecules, a lactone precursor C_22_H_32_O_12_ well described in sessile oaks was observed. It was also demonstrated in a previous study that this compound is a molecular marker of sessile oaks [26,38]. 

Cluster 9, also composed of molecules belonging to the triterpenoid family, is specific of sessile oaks with glycosylated terpenoids such as quercotriterpenoids [11,13]. Currently, around ten quercotriterpenoids have been identified. This cluster suggests the presence of other compounds of lower intensity, which may have related structures.

Clusters 10 and 11. The ions contained in these clusters are triterpenoid compounds: Cluster 10 contains bartogenic acid and oleanane derivatives and Cluster 11 contains triterpenes of larger size in the form of a dimer with the unitary brick of roburgenic or bartogenic acid. 

Molecular networks provide an overview of hydro-ethanolic extracts of sessile and pedunculate oaks by rapidly highlighting common compounds and species-specific ones. These results are consistent with previous studies that reported ubiquitous tannin derivatives in both species; a higher content in GluBA and terpene derivatives in pedunculate oaks, whereas sessile oaks contain more QTT and lactone precursors [24,25,26]. 

### 2.2. Species Assignment of an Unknown Sample 

In order to assess whether the molecular network could be used to determine the species of an unknown oak sample and thus be a tool to better identify a species on the basis of its molecular composition, extracts from individual oak trees were introduced into the molecular network and compared to the pooled samples of both species. The methodology was implemented to determine the species of 10 samples collected in various forests. The results presented illustrate the distribution of one pedunculate and one sessile oak individual, in the Venn diagram and in the network. Similar results were obtained for the other samples.

#### 2.2.1. Addition of Pedunculate Oak Individuals to the Network

**Figure 3:**
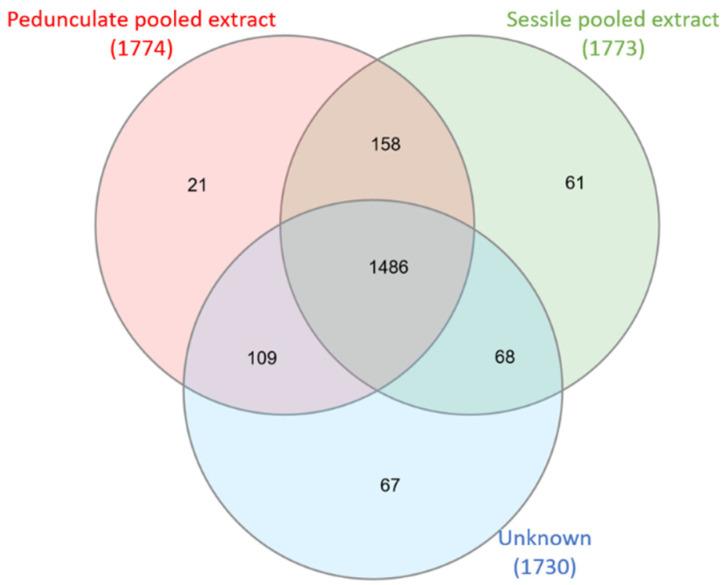
Venn diagram of an unknown individual sample (1 sample) attributed pedunculated compared to sessile pooled extracts (21 samples) and pedunculate pooled extracts (21 samples). Numbers above species represent total precursor ions detected in each extract. The circle overlap represents common ions, and the non-overlapping part represents specific ions.

First, a Venn diagram was built in order to compare the MS/MS data of the pedunculate oak individual with MS/MS data of pooled sessile and pedunculate oak samples (Figure 3).

Again, most of the ions are common for the three samples (1486). The number of ions shared only with the pedunculate oak pool (109) is greater than the number of ions shared with the sessile oak pool (68), in favor of a suitable assignment. Some ions (67) are specific to the individual, which can be explained by the high intra-individual variability even within the same species.

**Figure 4:**
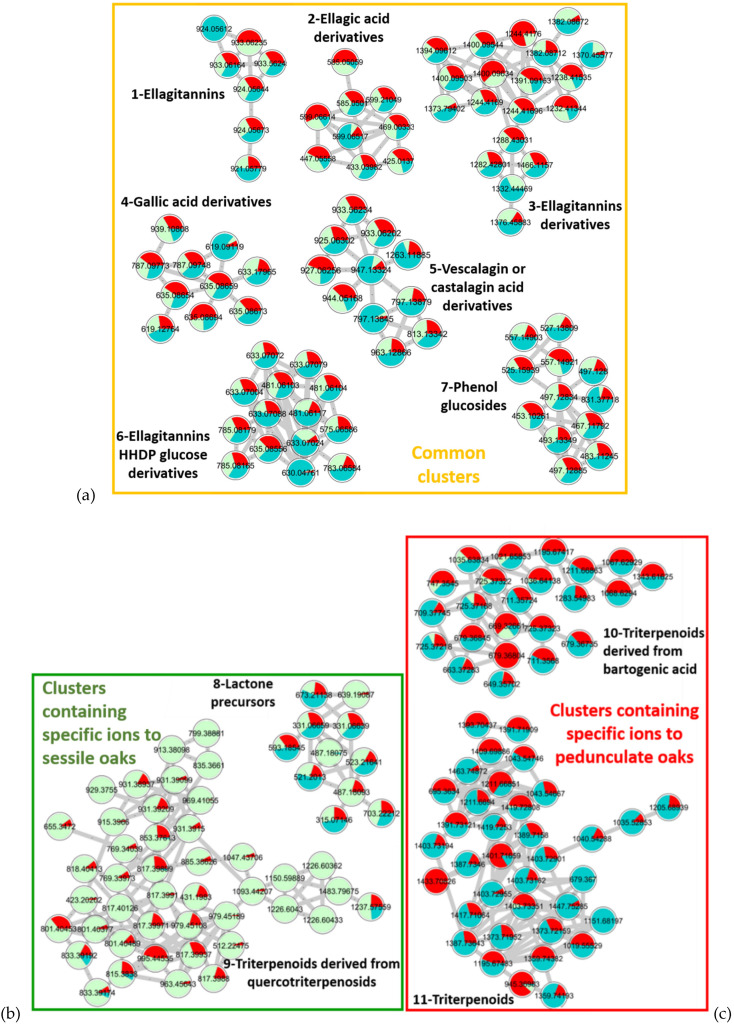
Molecular network representing the distribution of an individual (1 sample) of pedunculate oak (blue) compared to the pool (21 samples) of sessile (green) and (21 samples) pedunculate (red) oaks with cos 0.75 and matched fragment 6 parameters. (**a**) Clusters 1–7 representing common clusters shared between sessile, pedunculated and unknown individual, (**b**) Clusters 8 and 9 containing specific ions to sessile oaks and (**c**) Clusters 10 and 11 containing specific ions to pedunculated oaks shared with unknown individual.

A quantitative molecular network was again generated with addition of the individual to see how it was distributed in the network (Figure 4). 

On the one hand, the individual (in blue) is fairly distributed within the sessile pool and the pedunculate pool for the common Clusters 1 to 7 described in Table 1. On the other hand, the individual shares very few ions with triterpenoids derived from quercotriterpenosids, which are specific of sessile oaks in Cluster 9. It is also not distributed in Cluster 8 regarding the lactone precursors (*m/z* 487.1821 and 639.1908), which are also markers of the sessile oak pool. However, it is well distributed with the other ions of Cluster 8, which are shared between the two pools of species, highlighting the lack of selectivity of these compounds in the extracts compared to the lactone precursors. As for the two pedunculate-specific Clusters 10 and 11, the distribution is clearly visible across all nodes. Different relative abundances of ions are observed. Some molecules are fairly well shared between the pedunculate pool sample and the individual sample, whereas others are more abundant in either sample due to the specific chemical composition of the individual. Considering both the sharing of the pedunculate-specific molecules and the non-sharing sessile ones, this individual was assigned to the pedunculate species, which was confirmed by the genetic analyses of the individuals.

#### 2.2.2. Addition of Sessile Oak Individuals to the Network

**Figure 5:**
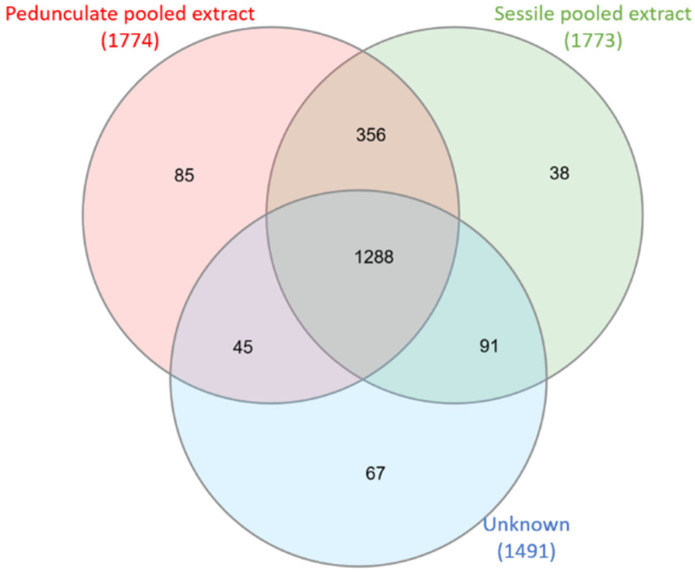
Venn diagram of an unknown individual sample (1 sample) attributed sessile compared to sessile pooled extracts (21 samples) and pedunculate pooled extracts (21 samples). Numbers above species represent total precursor ions detected in each extract. The circle overlap represents common ions, and the non-overlapping part represents specific ions.

In order to illustrate the method with a sessile oak individual, the same approach was implemented. The Venn diagram is shown in Figure 5.

It again highlights a majority of common ions (1288). The number of ions shared only with the sessile oak pool (91) is greater than the number of ions shared with the pedunculate oak pool (45).

**Figure 6:**
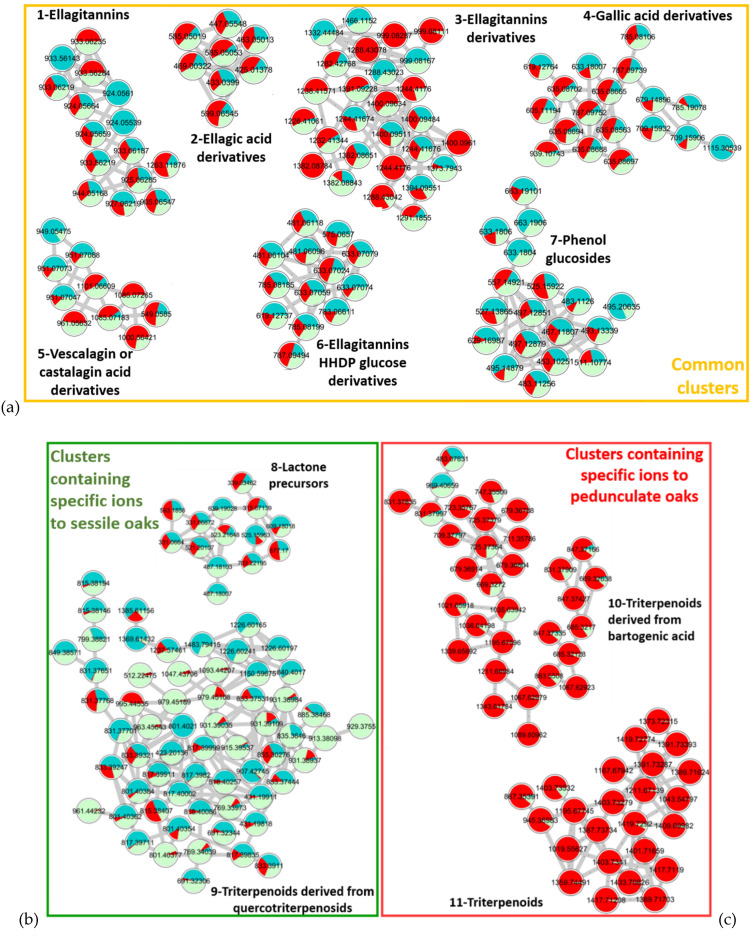
Molecular network representing the distribution of an individual (1 sample) of sessile oak (blue) compared to the pool (21 samples) of sessile (green) and (21 samples) pedunculate (red) oaks with cos 0.75 and matched fragment 6 parameters. (**a**) Clusters 1-7 representing common clusters shared between sessile, pedunculated and unknown individual, (**b**) Clusters 8 and 9 containing specific ions to sessile oaks shared with unknown individual and (**c**) Clusters 10 and 11 containing specific ions to pedunculated oaks.

The distribution of the individual in the network is shown in Figure 6. 

The individual (in blue) is well distributed within the sessile pool and the pedunculate pool in the common clusters (1 to 7) described in Table 1. The individual is not present in Clusters 10 and 11 that are specific to pedunculate oak (only 2 nodes out of the 32 in the cluster). On the contrary, it is distributed in Cluster 9 composed of the triterpenoids derived from quercotriterpenosid of the sessile oak pool and also in the few specific compounds (*m/z* 487.1821 and 639.1908) in Cluster 8. Thus, this sample was assigned to the sessile species which was confirmed by the genetic analysis. 

These two individuals illustrated the possibility to well determine both species of sessile and pedunculated oak. First, the Venn diagram points out twice more common ions between the individual sample and pooled extract of the suited species. Then, the network consolidates species assignation confirming that a large number of species-specific molecules are well detected in the sample. The molecular network makes it possible to validate this distribution on a large number of markers and not only on a few major molecules in contrast to methods using targeted compounds. 

## 3. Materials and Methods

### 3.1. Chemicals

Ethanol used for oak wood extraction was HPLC grade and was purchased from VWR (Fontenay-sous-Bois, France). Ultra-pure water was produced with a Purelab Flex system from Veolia (Wissous, France). Acetonitrile, water, and formic acid used for UHPLC-HRMS analysis were of Optima LC-MS grade from Fisher Scientific (Illkirch-Graffenstaden, France).

### 3.2. Oak Wood Extract Preparation

#### 3.2.1. Oak Wood Sampling

In order to constitute a representative oak extract which takes into account the intra-species variability which may be due in particular to the geographical location of the oaks, 20 samples were taken in 7 forests in the Centre-Val de Loire region in France. Thirty-two logs were also sampled at the cooperage (oak stave mill) on available wood in the timber yard with as many different woods as possible (growth ring widths/diameters). A wooden disk was cut from each freshly felled tree and the heartwood was collected.

#### 3.2.2. Genetic Analyses for Species Assignation

To distinguish oak species, genetic analyses were conducted by the CGAF-ONF laboratory (UMR BioForA, Orléans, France). For this study, total genomic DNA was extracted on the same trees on the same forest stands and cambial tissues from the same cooperage samples as previously, using the NucleoSpin^®^ 96 Plant II Kit (Macherey-Nagel, Hoerdt, France). Genotype data were obtained on the capillary of an ABI 3500 automatic sequencer (Applied Biosystems, Foster City, CA, USA) using 18 nuclear microsatellite markers developed by Guichoux et al. and analyzed with GeneMapper™ software v4.1 (Applied Biosystems) [45].

The determination of the species was carried out using an assignment method from Pritchard et al. using structured software. The method consists in comparing the multi-locus genotypes with the reference data for each species. If the percentage of assignment is above 80%, the sample is assigned to the species. In total, the sample set was composed of 26 pedunculate oaks and 26 sessile oaks: 21 were used to make pooled extracts and 5 were used as individuals (cf. supplementary data).

#### 3.2.3. Oak Wood Extraction

Two pooled extracts were prepared: one pooled extract of pedunculate oak containing the 21 pedunculate samples and another of sessile oaks was prepared with the same number of sessile samples.

The powdered mixture (2 g) of 21 oak wood samples was extracted using ultrasound-assisted extraction with 30 mL of water/ethanol mixture (85:15 *v*/*v*) during 1 h. The extract was then centrifuged at 5000× *g* during 5 min. The supernatant was recovered and analyzed.

Per species, 5 samples were used to demonstrate the distribution of unknowns introduced into the network. The 5 individuals of sessile oaks came from Aboncourt, Russy, Orléans, and Abbayes forests, while the 5 individuals of pedunculate oaks came from Sully la Chapelle, Chateauroux, Lisledon, and Abbayes forests.

### 3.3. UHPLC-HRMS/MS Analysis

Chromatographic analyses were performed using an Ultimate 3000 RSLC system equipped with an autosampler, a binary pump, a thermostated column compartment, and a DAD detector (Dionex, Germering, Germany). The column was a Luna^®^ Omega C18 (150 × 2.1 mm; 1.6 µm) (Phenomenex, Le Pecq, France). The mobile phase was composed of water (A) and acetonitrile (B), both acidified with 0.1% of formic acid. Elution was performed at a flow rate of 500 μL min^−1^ and with the following binary gradient program: starting with 3% of solvent B during 0.2 min, 3–45% B from 0.2 to 12 min, 45–90% B from 12 to 14 min, 90–3% B from 15 to 15.5 min. Then the column was re-equilibrated with 3% of solvent B during 3 min. The column temperature was set at 40 °C and 5 µL of the oak extracts were injected.

The MS and MS/MS experiments were carried out on a maXis UHR-Q-TOF mass spectrometer (Bruker, Bremen, Germany) with an electrospray ion source (ESI), working in negative ionization mode that allows for better detection and isolation of the molecular ion compared to the positive ionization mode. The pressure of nebulizing gas was set to 2 bar, and the flow rate and temperature of dry gas were set at 9.0 L min^−1^ and 200 °C. The capillary voltage was set at 4 kV. Mass spectra were summed during 400 ms in the *m/z* range 50–2250. All the MS data were processed using DataAnalysis software version 4.4 (Bruker). Molecular formulae were generated using the SmartFormula algorithm with an elemental composition of C, H, O to an infinite number and N ≤ 4 with a mass accuracy ≤3 ppm and were submitted to the SciFinder, PubChem, Lotus, and GNPS databases in order to propose compound structures.

MS/MS experiments were conducted using Data Dependent Acquisition mode with 3 precursor ions in the *m/z* range 150–1600; ions were excluded after 6 s. The analysis included 2 segments: in the first one, from 0 to 8 min, collision energies (CE) of 40 eV for monocharged ions and 30 eV for doubly charged ions were applied. In the second one, from 8 min to the end of the analysis, collision energies of 75 eV for monocharged ions and 40 eV for doubly charged ions were applied. CE values were adapted to the kinds of compounds: before 8 min, the molecules belong to the tannin family, which is mostly composed of C-O bonds, weaker than the C-C bonds of the terpenes, which elutes after 8 min. CE values for doubly charged ions are lower than for single-charged ones due to the proximity of both charges. MS/MS spectra were summed during 400 ms, so the total cycle time for MS and MS/MS was 1.6 s.

### 3.4. Venn Diagrams

A Venn diagram shows the logical relation between sets; it illustrates sample relationships by comparing common ions between them. InteractiVenn software (http://www.interactivenn.net/index2.html, accessed for the last figures on 30 September 2021) was used to build the Venn diagrams [46].

After analyses of the pooled extracts, sets were built using a bucket associating for each detected compound its retention time and the *m*/*z* of the most abundant ion. One set is represented by a circle, the overlap between two circles or sets is the ions they have in common, whereas the others are ions that are specific to a set.

### 3.5. Molecular Network Design

Molecular networks are visual representations of the chemical space present in tandem mass spectrometry (MS/MS) experiments by comparing mass spectra in pairs to map an extract. Therefore, considering that ions with closed structures will give similar neutral losses or fragment ions, each cluster associates molecules with a similar fragmentation pathway that possess high structural similarities and are likely to belong to the same chemical family.

The bucket table was built using MetaboScape software version 4.0 (Bruker). The T-ReX 3D algorithm detected precursor ions with an intensity threshold of 10,000 .a.u. and associated retention time, *m*/*z*, and area for each analyte.

The mascot generic format (mgf) file and a quantification table were exported to the GNPS platform (http://gnps.ucsd.edu, accessed for the last treatments on 7 and 8 September 2021) using feature-based molecular networking (FBMN) in order to build the molecular network [29,47]. Optimized parameters used for molecular network design were: mass tolerance 0.02 Da for parent and fragment mass, min pair cos 0.75, network TopK 10, maximum connected component size 100, minimum matched fragment ions 6, minimum cluster size 2, yes run MSCluster.

Quantitative molecular networks were visualized using Cytoscape software version 3.8.1 (https://cytoscape.org, accessed for the last figures on 7 and 8 September 2021). Within the network, one node corresponds to one MS/MS spectrum. Nodes are represented by pie charts. The proportion of each color in the nodes correlates with the relative abundance of the ion in each sample. The more abundant an ion is in a sample (species), the more dominant the color of this sample is in the pie chart.

## 4. Conclusions

The developed method based on an untargeted UHPLC-MS/MS analysis of several families of specialized metabolites of oak species (phenolic and terpenic compounds) was able to discriminate sessile and pedunculate chemical compositions. Both species showed specific molecules with similar fragmentation patterns associated in specific clusters in the molecular network. The comparison of individual unknown samples with pooled samples composed of extracts from several trees of pure species from various origins enabled the sample species to be assigned using the proposed two-step method: firstly, with the Venn diagram as a quick way to obtain an initial idea of how close an individual is to a species by comparing the number of similar ions detected in the sample vs. the pooled extracts that allow for the presumption of the sample species. Then with the molecular network, it is possible to visualize more precisely, at the level of specific clusters, the ions that are shared or not shared with one of the species. Due to the intra-species variability observed on individual samples, the confidence level of the species identification based on the detection of numerous compounds with related structures belonging to the same molecular family is improved, compared to the ones based on only few markers. Compared to genetic analysis, this method also presents advantages such as easier sample collection and conservation, higher convenience, and lower price. This method could now be applied on any new oak samples (one individual or a set of samples) to help in species identification. 

## Figures and Tables

**Table 1:** Description of the global network that compares pedunculate oak and sessile oak.

Cluster Number	R_T_ (min)	Measured *m/z* [M−H]^−^ or [M−2H]^2−^	Formula [M]	Error (ppm)	MS/MS Fragments [M−H]^−^	Proposed Annotation for Molecules	Molecular Family of the Cluster
1	4.10	933.0625^1−^	C_41_H_26_O_26_	−0.1	631.0582 C_27_H_19_O_18_ 300.9998 C_14_H_5_O_8_	Vescalagin or Castalagin [32]	Ellagitannins
	3.16	924.0571^2−^	C_82_H_50_O_51_	−1.4	631.0.581C_27_H_19_O_18_ 300.9993 C_14_H_5_O_8_	Roburin D or A [32]	
2	7.15	433.0401^1−^	C_19_H_14_O_12_	−1.5	300.9910 C_14_H_5_O_8_	Ellagic acid pentoside [33]	Ellagic acid derivatives
	7.40	447.0556^1−^	C_20_H_16_O_12_	0.8	300.9913 C_14_H_5_O_8_	Methyl ellagic acid pentoside [34]	
3	3.46	999.0811^2−^	C_111_H_172_O_31_	0.1	1065.1018 C_46_H_33_O_30_ 975.0744 C_43_H_27_O_27_ 933.0650 C_41_H_25_O_26_ 631.0575 C_27_H_19_O_18_	Grandinin or Roburin E derivative	Ellagitannin derivatives
	3.33	1227.4106^3−^	x	x	1065.1093 C_46_H_33_O_30_ 933.0631 C_41_H_25_O_26_ 915.0539 C_41_H_23_O_25_ 783.0690 C_34_H_23_O_22_ 631.0574 C_27_H_19_O_18_	Glycosylated grandinin	
4	6.26	635.0873^1−^	C_27_H_24_O_18_	−0.5	483.0678 C_20_H_19_O_14_ 465.0678 C_20_H_17_O_13_ 313.0568 C_13_H_13_O_9_ 169.0148 C_7_H_5_O_5_	Trigalloyl glucose [35]	Gallic acid derivatives
	7.16	939.1158^1−^	C_34_H_28_O_22_	−0.3	769.0892 C_34_H_25_O_21_ 617.0753 C_34_H_17_O_12_ 447.0573 C_20_H_15_O_12_ 169.0151 C_7_H_5_O_5_	Penta-galloylglucose [36]	
5	3.57	1101.0667^1−^	C_48_H_30_O_31_	−0.9	1057.0797 C_47_H_29_O_29_ 933.0605 C_41_H_25_O_26_ 631.0574 C_27_H_19_O_18_ 425.0141 C_20_H_9_O_11_	Vescavaloninic acid [37]	Vescalagin or castalagin acid derivatives
	3.71	961.0641^1−^	C_42_H_26_O_27_	−0.6	917.0683 C_41_H_25_O_25_ 873.0778 C_40_H_25_O_23_ 615.0631 C_27_H_19_O_17_ 491.062 C_25_H_15_O_11_	Deoxy-carboxy vescalagin [7]	
6	1.89	481.0611^1−^	C_20_H_18_O_14_	0.1	300.9951 C_14_H_5_O_8_ 275.0161 C_13_H_7_O_7_	HHDP glucose [34]	Ellagitannins HHDP glucose derivatives
	3.87	783.0662^1−^	C_34_H_24_O_22_	−0.6	481.0640 C_20_H_17_O_14_ 300.9994 C_14_H_5_O_8_	Pedunculagin [36]	
7	7.30	497.1289^1−^	C_22_H_26_O_13_	−0.9	313.0574 C_13_H_13_O_9_ 183.0655 C_9_H_11_O_4_ 169.0136 C_7_H_5_O_5_	3,4,5-trimethoxyphenyl-(6′-O-galloyl)-O-*β*-glucopyranoside [38]	Phenol glucosides
	5.58	453.1027^1−^	C_20_H_22_O_12_	0.6	313.0569 C_13_H_13_O_9_ 327.0706 C_14_H_15_O_9_ 183.0293 C_8_H_7_O_5_ 169.0152 C_7_H_5_O_5_	3-methoxy-4-hydroxyphenol 1-O-*β*-d-(6′-O-galloyl)glucopyranoside [39]	
8	9.01	487.1821^1−^	C_22_H_32_O_12_	0.1	211.0266 C_9_H_7_O_6_ 168.0074 C_7_H_4_O_5_^●^ 124.0233 C_6_H_4_O_3_^●^	3-Methyl-4-[[6-O-(3,4,5-trihydroxybenzoyl)-*β*-d-glucopyranosyl]oxy] octanoic acid [40,41,42]	Lactone precursors
	10.40	639.1908^1−^	C_29_H_36_O_16_	−0.2	271.0454 C_11_H_11_O_8_ 211.0244 C_9_H_7_O_6_ 169.0145 C_7_H_5_O_5_ 125.0249 C_6_H_5_O_3_	Not identified	
9	10.60	817.3991^1−^	C_43_H_62_O_15_	−0.6	655.3483 C_37_H_51_O_10_ 611.3593 C_36_H_51_O_8_ 169.0139 C_7_H_5_O_5_	Quercotriterpenosid [11,12]	Triterpenoids derived from quercotriterpenosids
	9.95	979.4518^1−^	C_49_H_72_O_20_	−0.6	817.4011 C_43_H_61_O_15_ 755.4010 C_42_H_59_O_12_	Quercotriterpenosid derivative [11,12]	
10	10.90	679.3680^1−^	C_36_H_56_O_12_	−0.5	517.3207 C_30_H_45_O_7_ 455.3171 C_29_H_43_O_4_ 437.3075 C_29_H_41_O_3_	Glucosylated bartogenic acid [43,44]	Triterpenoids derived from bartogenic acid
	14.30	669.3272^1−^	C_37_H_50_O_11_	−2.8	517.3173 C_30_H_45_O_7_ 455.3171 C_29_H_43_O_4_ 437.3060 C_29_H_41_O_3_	Galloyl bartogenic acid [43,44]	
11	13.50	1373.7248^1−^	C_72_H_110_O_25_	−0.1	695.3649 C_36_H_55_O_13_ 647.3443 C_35_H_51_O_11_ 485.2909 C_29_H_41_O_6_ 471.3113 C_29_H_43_O_5_	Roburosid B or C [43,44]	Triterpenoids
	13.80	1403.7341^1−^	C_72_H_110_O_24_	0.2	679.3705 C_36_H_55_O_12_ 517.3173 C_30_H_45_O_7_	Roburosid A [43,44]	

## Data Availability

The data presented in this study are available on request from the corresponding author. The data are not publicly available due to [industrial partnerships of the program].

## Supplementary Materials

The following are available online at https://www.mdpi.com/article/10.3390/metabo11100684/s1, Table S1: Composition of sessile pooled extract, Table S2: Composition of sessile pooled extract, Table S3: Listing of individual samples, Figure S1: Representative MS/MS spectra of each cluster.
