# Supplementary material for: Oak Species Quercus robur L. and Quercus petraea Liebl. Identification Based on UHPLC-HRMS/MS Molecular Networks
Source: Metabolites. 2021 Oct 6;11(10):684. doi: 10.3390/metabo11100684 (PMC8540037; doi:10.3390/metabo11100684)
Supplement: Supplementary file 1 [file metabolites-11-00684-s001.zip › metabolites-1396810-supplementary.pdf]

<u>Supplementary data</u>

Species assignments according to the DNA analyses

Qro: *Quercus robur* (pedunculate oak), Qpe: *Quercus petraea* (sessile oak), Qpy: *Quercus pyrenaica* (tauzin oak), Qpub: *Quercus pubescens* (pubescent oak)

- *Table S1: Composition of sessile pooled extract*

Location	Pedunculate Qro	Tauzin Qpy	Pubescent Qpub	Sessile Qpe	Species
**Aboncourt**	0.004	0.002	0.006	0.987	Qpe
**Amboise**	0.009	0.010	0.004	0.978	Qpe
**Polisy**	0.005	0.004	0.007	0.985	Qpe
**Sully la chapelle**	0.003	0.003	0.007	0.988	Qpe
**Aboncourt**	0.146	0.010	0.011	0.834	Qpe
**Fontainebleau**	0.004	0.005	0.139	0.852	Qpe
**Dainville-Bertheléville**	0.051	0.005	0.010	0.933	Qpe
**Saint Désiré and Saint Eloy d'Allier**	0.004	0.002	0.018	0.976	Qpe
**Igé**	0.095	0.002	0.042	0.862	Qpe
**Azé**	0.008	0.007	0.005	0.979	Qpe
**Blois**	0.004	0.003	0.003	0.989	Qpe
**Compiègne**	0.004	0.020	0.006	0.971	Qpe
**Asnières sous bois**	0.005	0.003	0.005	0.988	Qpe
**Villedieu sur Indre**	0.003	0.024	0.012	0.960	Qpe
**Etréchy**	0.003	0.002	0.003	0.992	Qpe
**Cellettes**	0.004	0.006	0.006	0.983	Qpe
**Le Moulinet sur solin**	0.004	0.008	0.004	0.984	Qpe
**Ingrannes**	0.002	0.002	0.005	0.990	Qpe
**Chalette sur loing**	0.092	0.016	0.036	0.856	Qpe
**Chevillon sur Huillard**	0.012	0.012	0.011	0.965	Qpe
**Poinçonnet**	0.004	0.003	0.003	0.99	Qpe

- *Table S2: Composition of pedunculate pooled extract*

Location	Pedunculate Qro	Tauzin Qpy	Pubescent Qpub	Sessile Qpe	Species
**Sully la chapelle**	0.987	0.007	0.005	0.978	Qro
**Bride**	0.992	0.003	0.003	0.003	Qro
**Bride**	0.973	0.019	0.003	0.005	Qro
**Dainville-Bertheléville**	0.983	0.003	0.007	0.006	Qro
**Igé**	0.988	0.006	0.003	0.003	Qro
**Azé**	0.889	0.063	0.028	0.019	Qro
**Compiègne**	0.984	0.005	0.005	0.006	Qro
**Villedieu sur Indre**	0.989	0.004	0.004	0.002	Qro
**Azé**	0.989	0.005	0.003	0.003	Qro
**Sully la chapelle**	0.986	0.003	0.006	0.005	Qro
**Polisy**	0.866	0.056	0.046	0.031	Qro
**Compiègne**	0.933	0.015	0.029	0.023	Qro
**Villedieu sur Indre**	0.990	0.003	0.004	0.003	Qro
**Azé**	0.980	0.008	0.007	0.005	Qro
**Etréchy**	0.971	0.005	0.010	0.014	Qro
**Arthon**	0.942	0.041	0.011	0.005	Qro
**Chevillon sur Huillard**	0.958	0.008	0.021	0.013	Qro
**Chevillon sur Huillard**	0.989	0.005	0.004	0.003	Qro
**Arthon**	0.896	0.011	0.007	0.086	Qro
**Chevillon sur Huillard**	0.993	0.003	0.002	0.002	Qro
**Poinçonnet**	0.95	0.024	0.018	0.008	Qro

- *Table S3: Listing of individual samples*

Location	Pedunculate Qro	Tauzin Qpy	Pubescent Qpub	Sessile Qpe	Species
**Aboncourt**	0.003	0.003	0.004	0.991	Qpe
**Orléans -Massif ingrannes**	0.002	0.002	0.005	0.990	Qpe
**Forêt des abbayes**	0.003	0.002	0.003	0.992	Qpe
**Forêt d'Orléans**	0.004	0.008	0.004	0.984	Qpe
**Rusy**	0.003	0.004	0.004	0.989	Qpe
**Chateauroux**	0.981	0.004	0.006	0.010	Qro
**Forêt des abbayes**	0.970	0.005	0.010	0.014	Qro
**Sully la chapelle**	0.988	0.004	0.004	0.004	Qro
**Chateauroux**	0.988	0.003	0.005	0.004	Qro
**Lisledon**	0.993	0.003	0.002	0.002	Qro

Description of the characteristic fragments used to annotate the molecular network

- *Figure S1: Representative MS/MS spectrum of each cluster*

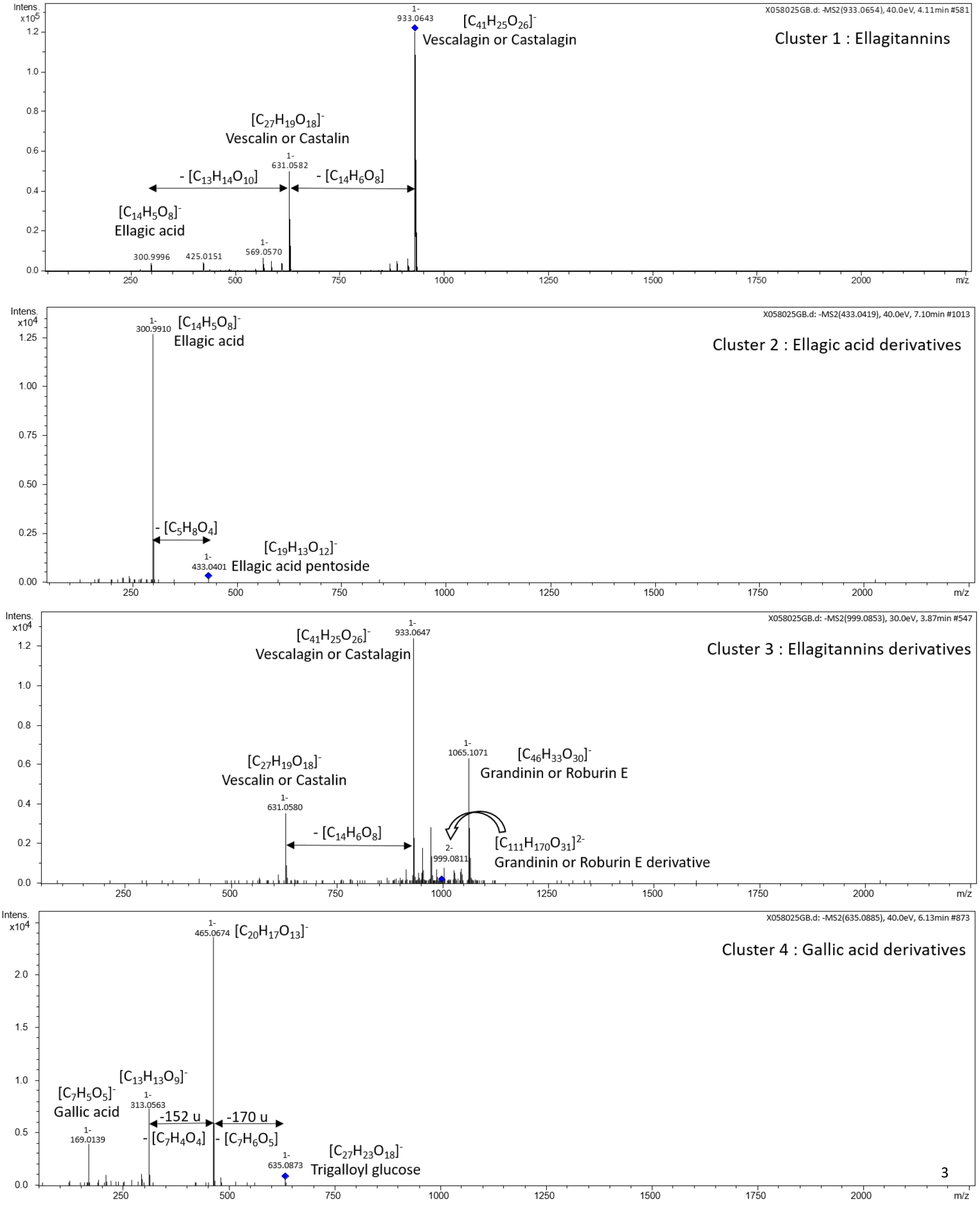

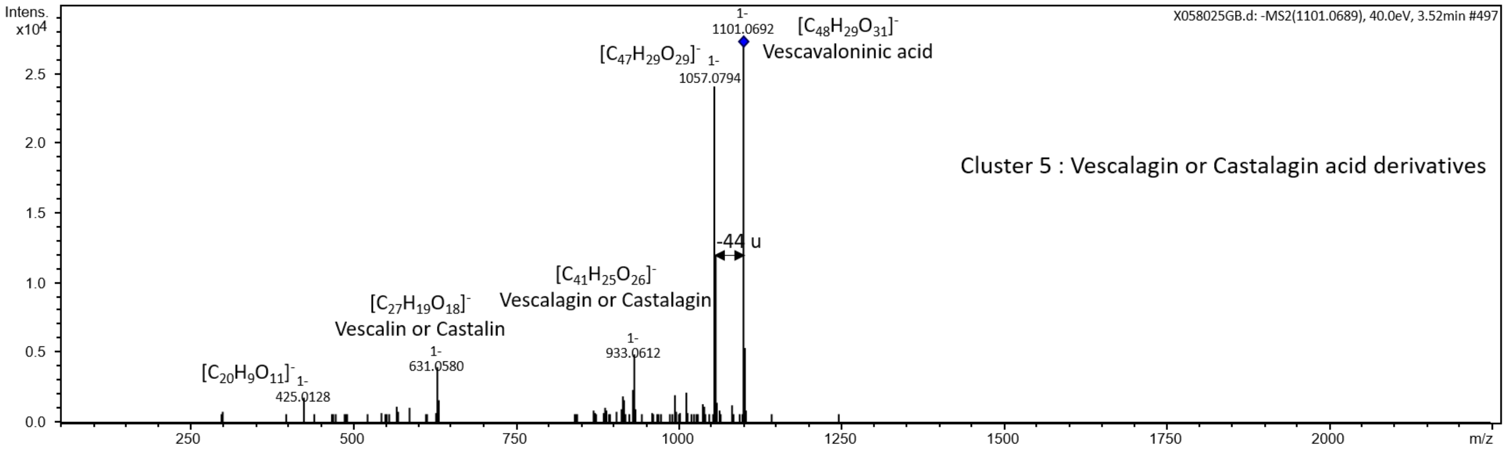
Intens. x10^4
X058025GB.d: -MS2(1101.0689), 40.0eV, 3.52min #497
Cluster 5 : Vescalagin or Castalagin acid derivatives
[C48H29O31]- Vescavaloninic acid
1- 1101.0692
[C47H29O29]- 1- 1057.0794
-44 u
[C41H25O26]- Vescalagin or Castalagin
1- 933.0612
[C27H19O18]- Vescalin or Castalin
1- 631.0580
[C20H9O11]- 1- 425.0128
m/z

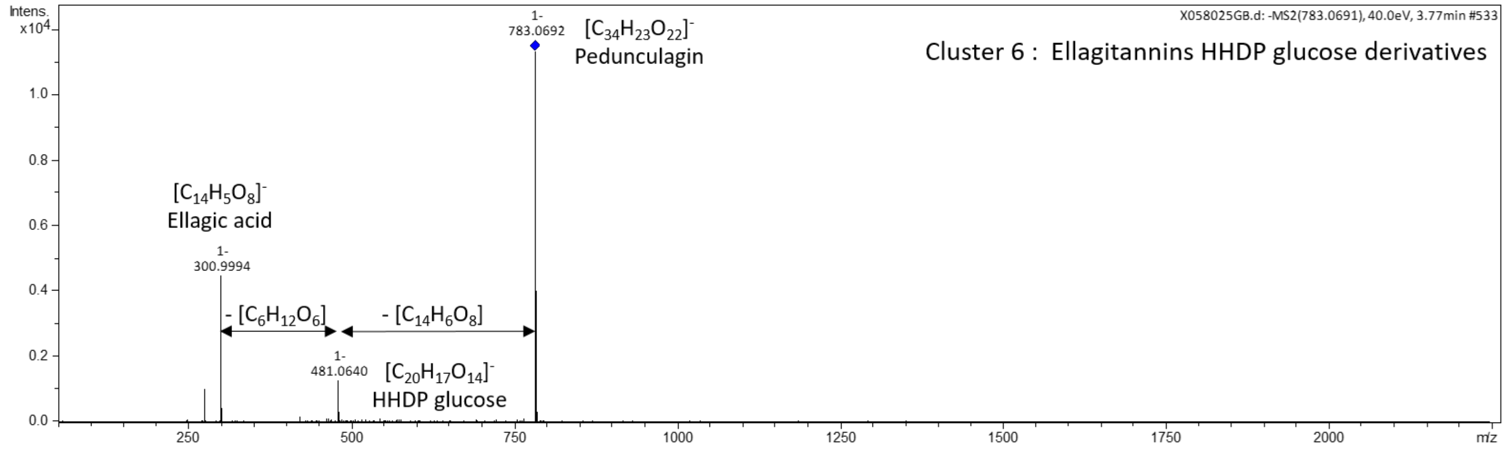
Intens. x10^4
X058025GB.d: -MS2(783.0691), 40.0eV, 3.77min #533
Cluster 6 : Ellagitannins HHDP glucose derivatives
1- 783.0692
[C34H23O22]- Pedunculagin
[C14H5O8]- Ellagic acid
1- 300.9994
- [C6H12O6]
- [C14H6O8]
1- 481.0640
[C20H17O14]- HHDP glucose
m/z

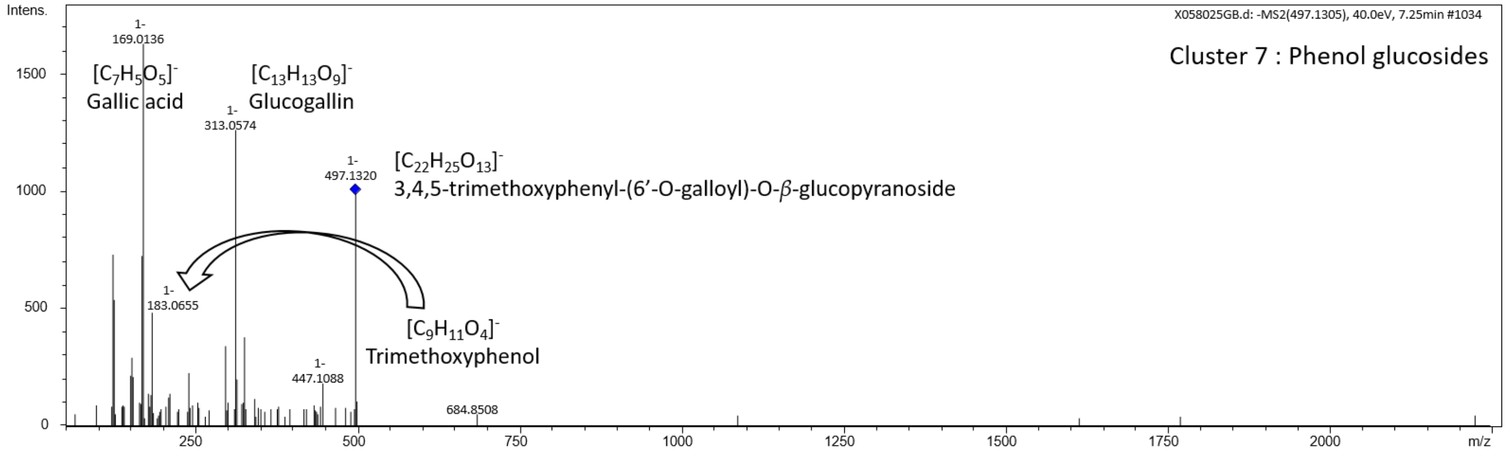
Intens.
X058025GB.d: -MS2(497.1305), 40.0eV, 7.25min #1034
Cluster 7 : Phenol glucosides
1- 169.0136
[C7H5O5]- Gallic acid
[C13H13O9]- Glucogallin
1- 313.0574
1- 497.1320
[C22H25O13]- 3,4,5-trimethoxyphenyl-(6'-O-galloyl)-O-β-glucopyranoside
1- 183.0655
[C9H11O4]- Trimethoxyphenol
1- 447.1088
684.8508
m/z

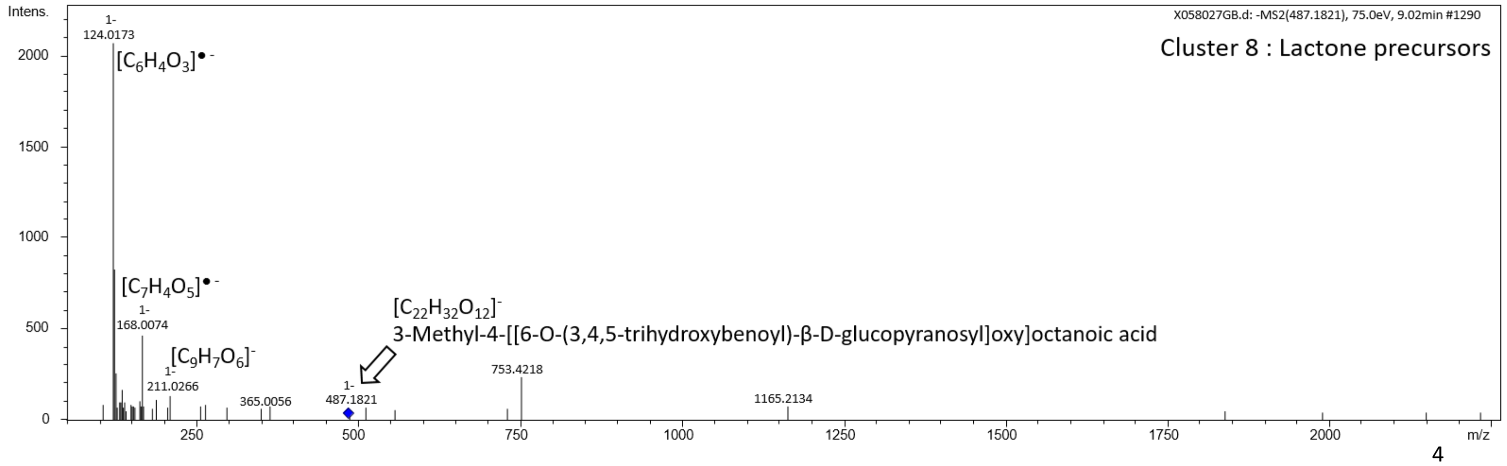
Intens.
X058027GB.d: -MS2(487.1821), 75.0eV, 9.02min #1290
Cluster 8 : Lactone precursors
1- 124.0173
[C6H4O3]•-
[C7H4O5]•-
1- 168.0074
[C9H7O6]-
1- 211.0266
365.0056
1- 487.1821
[C22H32O12]- 3-Methyl-4-[[6-O-(3,4,5-trihydroxybenoyl)-β-D-glucopyranosyl]oxy]octanoic acid
753.4218
1165.2134
m/z

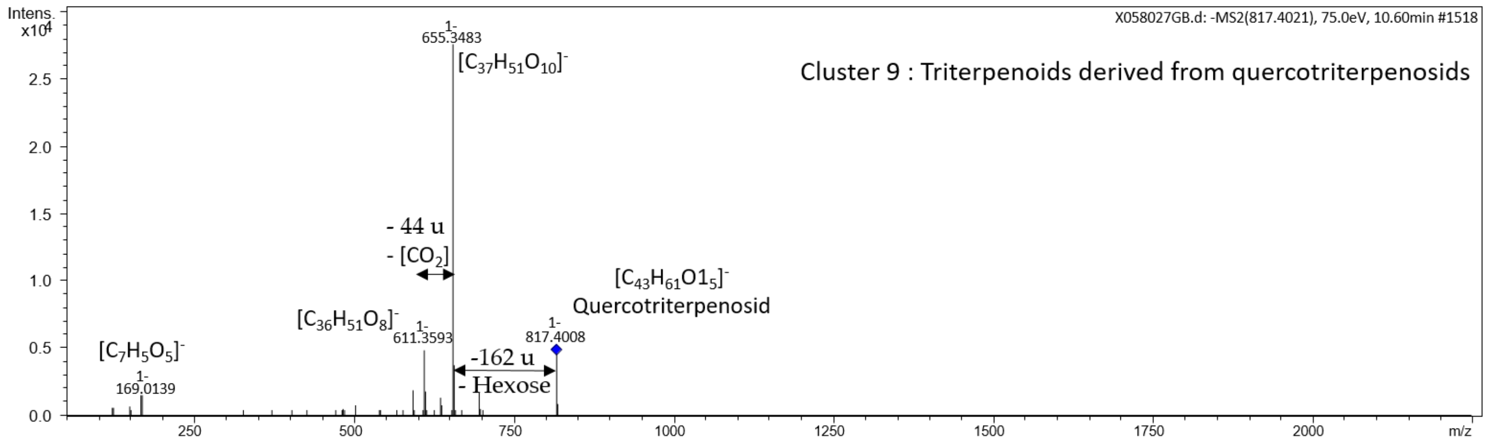
X058027GB.d: -MS2(817.4021), 75.0eV, 10.60min #1518
Cluster 9 : Triterpenoids derived from quercotriterpenosids
Intens. x10^4
655.3483
[C37H51O10]-
- 44 u
- [CO2]
[C43H61O15]-
Quercotriterpenosid
[C36H51O8]-
611.3593
817.4008
[C7H5O5]-
169.0139
-162 u
- Hexose
m/z

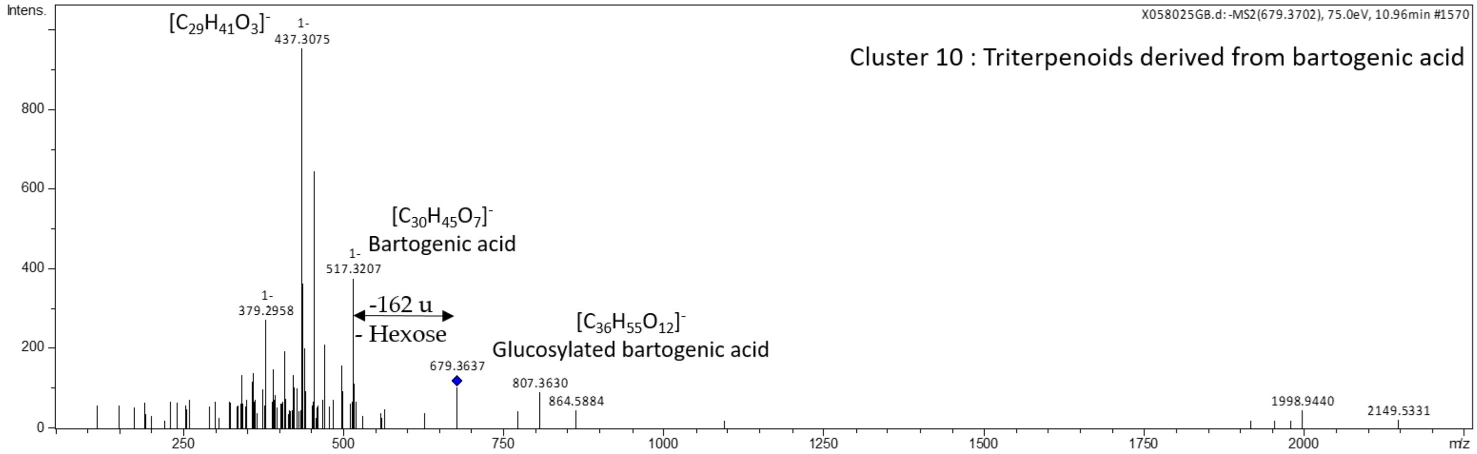
X058025GB.d: -MS2(679.3702), 75.0eV, 10.96min #1570
Cluster 10 : Triterpenoids derived from bartogenic acid
Intens.
[C29H41O3]-
437.3075
[C30H45O7]-
Bartogenic acid
517.3207
379.2958
-162 u
- Hexose
[C36H55O12]-
Glucosylated bartogenic acid
679.3637
807.3630
864.5884
1998.9440
2149.5331
m/z

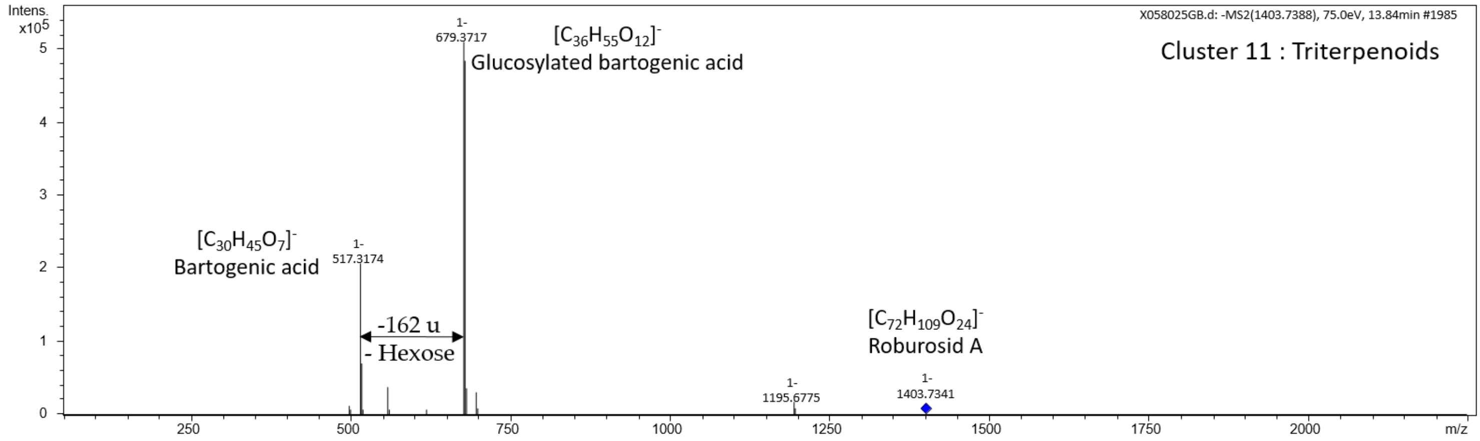
X058025GB.d: -MS2(1403.7388), 75.0eV, 13.84min #1985
Cluster 11 : Triterpenoids
Intens. x10^5
679.3717
[C36H55O12]-
Glucosylated bartogenic acid
[C30H45O7]-
Bartogenic acid
517.3174
-162 u
- Hexose
[C72H109O24]-
Roburosid A
1195.6775
1403.7341
m/z
